# Changes in Fish Consumption Desire and Its Factors: A Comparison between the United Kingdom and Singapore

**DOI:** 10.3390/foods7070097

**Published:** 2018-06-26

**Authors:** Atin Supartini, Taro Oishi, Nobuyuki Yagi

**Affiliations:** 1 Contract Research Organization (CRO) Department, Iberica Co., Ltd., 2-18-30 Hakataekihigashi, Hakata-ku, Fukuoka 812-0013, Japan; zamzabila@hotmail.com; 2 Faculty of Socio-Environmental Studies, Fukuoka Institute of Technology, 3-30-1, Wajiro-higashi, Higashi-ku, Fukuoka 811-0295, Japan; 3 Graduate School of Agricultural and Life Sciences, The University of Tokyo, 1-1-1, Yayoi, Bunkyo-ku, Tokyo 113-8657, Japan; yagi@fs.a.u-tokyo.ac.jp

**Keywords:** fish consumption change, questionnaire survey, the United Kingdom, Singapore

## Abstract

It is widely known that rapid population growth and income improvement in some developing countries, and growing health consciousness in some developed countries, are the main factors that have contributed to the global increase in the consumption of fishery products in the past few decades. While a detailed analysis of fish consumption behavior is being conducted in several countries, there are other countries where changes in fish consumption desire and their social and psychological factors are not fully clarified. This study investigates the changes in fish consumption desire and its associated factors in the United Kingdom (UK) and Singapore. Primary data were gathered from 1200 participants through a web questionnaire survey in the UK and Singapore. The results show that approximately five times as many respondents stated that the desire to purchase fish had increased in both the UK and Singapore compared to those that believed it had not 10 years ago. Second, the increase in fish consumption in the UK is positively associated with younger age, cheaper price, the health benefits of fish, concern over the health of meat, and religion. Third, the increase in fish consumption in Singapore is positively associated with cheaper prices and is negatively associated with concerns over sustainability.

## 1. Introduction

The total seafood consumption in the world has consistently increased over the past 50 years. The improvement of living standards in developing countries, such as China, and the health consciousness of some developed countries have contributed to this increase in consumption [1]. On the other hand, some countries of the former Soviet Union or the sub-Saharan African region have experienced a significant decrease in fish consumption over the recent decades [2]. Various factors are considered to be related to seafood consumption in each country. Importantly, the progress of globalization and increased interest in health consciousness and sustainability in recent years has the potential to cause drastic changes in fish consumption even within the same country.

Until now, related literature has analyzed fish consumption behavior and its factors. Understanding factors associated with fish consumption is crucial to implementing successful strategies to increase fish consumption in one’s own country or to export fish products to other target countries. According to the researcher who applied the Theory of Planned Behavior (TPB), attitudes, subjective norms, past experience, and health are the main factors that determine the intention and frequency of fish consumption [3]. This theory is acknowledged for its parsimony and ability to generalize across situations, behaviors, objects, individuals, and cultural settings. For example, Verbeke and Vackier [4] investigated the determinants of fish consumption behavior in Belgium using TPB and found that fish consumption is higher among women and increases with age, while it is lower in lower income classes. A higher level of education results in a greater intention to consume fishery products. Olsen et al. [5] stated that there is a difference in preference and motivation to consume products between younger and older consumers in Spain. The study by Kaimakoudi et al. [6] showed that high-potential fishery consumers are younger, earn a higher income, and have a higher educational background amongst Greek customers. In addition, consumers characterized by a healthy lifestyle are more likely to have healthy diets composed of fish in the U.S [7].

Many factors affect fish consumption behavior, and they vary between countries and between cultures as a result of the availability of commodities and economic factors [6]. We investigate the change in fish consumption desire and its associated factors in the United Kingdom (UK). In the UK, the government-appointed industry expert working group members suggest a strategic framework to drive growth in England’s seafood consumption up to 75%, from the current period to 2040 [8]. Therefore, it is meaningful to understand fish consumption factors in the UK in terms of policy. The Marine Stewardship Council (MSC) label (one of the most popular fishery ecolabels worldwide) was created in the UK; this label has penetrated rapidly and achieved recognition [9,10], and might be attributed to the increased fish consumption among Britishers. As a comparison to the UK, this study also investigates the change in fish consumption desire and its associated factors in Singapore; it is used as a representation of developed countries in Asia. It is observed that the consumption of fish and seafood among Singaporeans is more than the global average [11].

Overall, the aims of the present study are as follows: (i) to obtain information on the change in fish consumption pattern, and (ii) to investigate the influence of the social and psychological determinants on the change in fish consumption pattern. It is expected that the results of this research will assist any country, such as Japan (where the marine product market has been suffering due to a slump in domestic fishery consumption) in the decision-making process regarding the strategy to increase fish consumption.

## 2. Materials and Methods

We developed the contents of the questionnaires and online surveys by using the questionnaire conducted by a third-party survey company from 11–19 January, 2017, in the UK and Singapore. Six hundred individuals were selected from each country using stratified random sampling by age and gender, proportioning the ratios of gender and age to those of the respective country’s general population. We limited the sample to respondents between 20 and 69 years of age to avoid biased sampling of the elderly because there are many elderly who do not use the Internet (individuals aged 70 years or older who are comfortable with computer and Internet usage can be concentrated in a specific social group, such as those having a high educational history; therefore, this sample does not reflect the elderly population aged 70 years and above).

Table 1 shows a summary of the survey questionnaire for our main analysis—the multiple logistic regression. The participants responded about their fish consumption tendencies compared to 10 years previously by choosing one of the three options (increased, decreased, and unchanged). A form of questioning that reminds the respondents of a situation that existed 10 years ago or more has limitations in accuracy, but it has been implemented in several previous studies as a practical way of comparing different time points. For example, Jorm [12] reminded respondents of situations about their family and friends 10 years ago and compared it with present situations. Smith et al. [13] also reminded respondents of their situations 10 years ago and compared it with their present situations. The survey by Reilly et al. [14] also reminded respondents of a situation that occurred more than 20 years ago. In this research, our purpose is not to know about events that occurred 10 years ago, but to know about the change in the last 10 years, and hence we adopted the same question method as the one adopted in the aforementioned studies.

In addition, to investigate the factors related to changes in fish consumption, the participants were asked to respond about their current perspective on fish/seafood compared to what it had been 10 years previously by choosing from three options (closer to A, between A and B, closer to B). The method of asking whether the opinion of respondents is closer to A or B is adopted in the International Social Survey Program (ISSP) and is being followed in dozens of countries; we followed this method in the study. The options in group A are as follows: Fish has become cheaper, household income has increased, fish is good for health, eating meat is dangerous for health, fish variety has increased, fish produced in sustainable fisheries has increased, cooked products have increased, and “opt out of eating out” has increased. The options included in group B are as follows: Fish has become expensive, household income has decreased, fish is not good for health, eating meat is good for health, fish variety has decreased, fish produced in sustainable fisheries has decreased, cooked product has decreased, and “opt out of eating out” has decreased. The questions examine the effect of economic factors on fish consumption, such as fish prices and consumer income, changes in the demand for fish substitute goods (such as meat) and social factors (such as increased interest in fish nutrition, an increase in the variety of fish available, and an increased willingness to purchase fish owing to better sustainable fishery certification). An increase in the number of vegetarians is also considered to be a factor that has increased the consumption of fish, but the reason for becoming vegetarian overlaps with other factors considered in this study, such as health and the environment [15]. To prevent multiple collinearity between explanatory variables, we did not consider vegetarians in this study. The socio-demographic characteristics included in the analysis are age, sex, educational level, religion, and household composition.

We also asked the following question to supplement to the multiple logistic regression analysis: preference for sushi (Question: “Do you like sushi?” Answer option: Like very much, Like, Neither like nor dislike, Dislike, Dislike very much, and Never tried). Although sushi represents only one type of fish consumption, it has gained prominence over the years; the rapid spread of sushi may be associated with a change in fish consumption.

## 3. Statistical Analysis

First, we compared and analyzed the differences in socio-demographic characteristics and dependent variables between Singapore and the UK. Here, continuous variables were expressed as a mean ± SD, and the categorical variables were expressed as percentages. The differences between each country were assessed using Student’s t-test for continuous variables and a Chi square test (χ2) for categorical variables. A *p*-value of *p* ≤ 0.05 was regarded as statistically significant.

Second, multiple logistic regression analyses for each country were used to analyze the association between fish consumption and its associated factors. In the analyses, we examined the factors that explain the socio-demographic characteristics and health behavior of consumers. The dependent variable took the value of one if there was an increase in a consumer’s desire to eat fish, and zero if it did not change significantly.

All data analyses were conducted using Statistical Analysis System (SAS) version 9.2 (SAS Institute, Inc., Cary, NC, USA).

## 4. Results

The socio-demographic characteristics and health behaviors of the study population are stratified by country, as shown in Table 2. The mean age of the study subjects is 43.7 ± 13.4 years. The UK has a higher mean age of 44.1 ± 13.9 years when compared to that of Singapore. The distributions of several socio-demographic characteristics and health behaviors differed by country. Significant differences are evident in the desire to eat fish (*p* < 0.003), religion (*p* < 0.0001), education level (*p* < 0.0001), and household composition (*p* < 0.0001). The results show that the religion of most respondents (over 80%) in the UK is Christianity, while the respondents from Singapore are affiliated with several religions.

In general, the results show that the desire for fish consumption has increased in both the UK (by 40.3%) and Singapore (by 47.7%).

Table 3 presents the results of the multiple logistic regression analysis. In this case, the odds ratio (OR) index shows the likelihood of an event in the two groups; additionally, the results show that the larger the index becomes, the stronger the desire becomes to consume fish. Table 3 shows that there is a 95% confidence for the population mean to be within the range. Additionally, when the confidence interval (CI) is more than 1, there is a positive and significant association; when the CI is less than 1, there is a negative and significant association.

The results show that in the UK, an increase in the desire for consuming fish is significantly associated with younger age (OR: 6.80, 95% CI: 2.99–15.43), the price of the fish (OR: 3.44, 95% CI: 1.73–6.93), the health benefits of fish (OR: 3.67, 95% CI: 1.46–9.21), concern over the health of meat (OR: 2.82, 95% CI: 1.24–6.41), and religion (OR: 1.03, 95% CI: 1.01–1.05).

In Singapore, the relative low cost of fish is significantly associated with an increase in the desire for consuming fish (OR: 5.00, 95% CI: 2.12–11.77). Reduced opportunities to buy sustainable fishery products are less likely to increase the desire for consuming fish (OR: 0.49, 95% CI: 0.25–0.98), which is consistent with expected results. On the other hand, both the health benefits of fish (OR: 0.42, 95% CI: 0.19–0.95) and the health risk of fish (OR: 0.35, 95% CI: 0.15–0.80) have negative impacts on an increase in the desire for consuming fish. The interpretation of these results is difficult and may be related to future research subjects mentioned at the end of this study.

**Figure 1:**
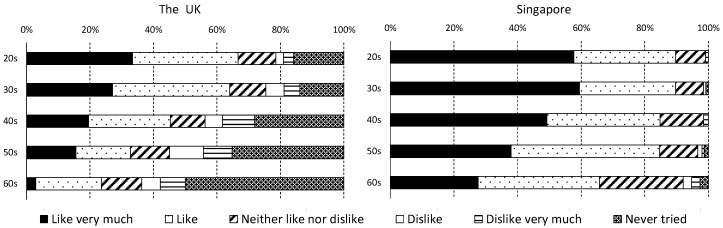
Answers to the question “Do you like sushi?”.

Figure 1 shows the results of a supplementary question (“Do you like sushi?”) asked in this questionnaire survey. Overall, the results show that consumers in Singapore prefer more sushi than those in the UK, and the generation gap between consumers who like sushi (the sum of the percentage of “Like very much” and “Like”) is wider in the UK than it is in Singapore. Moreover, in Singapore, there are a few people who have never eaten sushi in any age; however, in the UK, the percentage of “Never tried” is in the higher age bracket.

## 5. Discussion

The implications of the results are as follows:

First, approximately five times more respondents in the UK and Singapore stated that their desire to eat fish has increased, compared to what it had been 10 years previously (see Table 2). These countries are developed countries that are triggering an increase in fish consumption and are becoming more important as targets for fishery export countries.

Second, an increase in the desire to consume fish in the UK is associated with younger age, while no significant relationship was found in Singapore. In the UK, it can be thought that fish diets have sharply increased due to changes in the food habits of young people. As seen in Figure 1, in Singapore, there are few people who have never eaten sushi in any age; however, in the UK, the percentage of “Never tried” is in the higher age bracket. There is a possibility that the adoption of sushi by the young consumer population shows a difference in food preferences between young and older consumers, and hence represents the generational gap in food preferences. This gap has led to a change in the fish consumption scenario in the UK. The adoption of sushi by the older population of consumers is further expected to increase fish consumption in the UK.

Third, relative cheapness is an important factor that has contributed to an increase in fish consumption in both countries. A previous study conducted in the north and northeast of Thailand revealed that pricing is the most critical factor affecting the decision to purchase fishery products [16]. We found that pricing is a major determinant of fish consumption, not only in developing countries like Thailand, but also in developed countries like the UK and Singapore.

Fourth, in the UK, health consciousness has been a factor that has contributed to an increase in fish diets, as suggested in past studies to date, but clear results have not been obtained in Singapore. According to TPB, attitudes (e.g., eating fish is healthy, and safety), social norms (e.g., preparing healthy food for the family), and health perspectives are the main determinants of food consumption behavior, including fish and seafood [5]. Similarly, we find that health-related concerns (fish is healthy, meat is unsafe) are significantly associated with fish consumption in the UK, but not in Singapore. Furthermore, it was found that the perceptions that fish is good for health, as well as the concern about the safety of meat (which serves as a substitute to fish), are affecting fish consumption in the UK. Considering that Bovine Spongiform Encephalopathy (BSE) became a big problem in the UK and did not occur in Singapore [17], the safety of beef due to BSE still poses a concern in the UK, and might be one of the reasons for an increase in the fish diet.

Fifth, in Singapore, an increase in the desire to consume fish is negatively associated with a concern about sustainability. An emphasis on sustainable fishery practices is increasingly becoming a crucial factor affecting fish consumption in Singapore, which will need to be considered when other countries export fish products to Singapore. However, according to an interview survey conducted as part of the project related to this research, almost no fisheries that are certified as sustainable by an ecolabel are displayed in Singapore’s supermarkets, and the awareness of local consumers about this ecolabel is low. Thus, Singapore must promote the ecolabel. On the other hand, the Marine Stewardship Council (MSC) label (one of the most popular fishery ecolabels worldwide) was created in the UK; this label has penetrated rapidly and achieved recognition [9,10]; however, the popularity of the ecolabel is not associated with increased fish consumption in the UK.

Finally, our findings show that religion is significantly associated with an increase in fish consumption in the UK, but not in Singapore. Daniel [18] states that food is an important part of a culture and serves economic, social, and religious functions. This finding coincides with the findings of previous research. As we mentioned in the results section, most respondents who answered they were religious were Christian in Britain. According to Assadi [19], “one can expect a sharp decrease in the sale of beef and red meat, which can be easily compensated by the increase in fish, seafood and chicken sales that substitute beef” during the 40 days in which Lent is celebrated among the Catholics. There is a possibility that Lent is related to the reason why the estimated value of the coefficient of religion was significant in the UK.

## 6. Conclusions

According to the obtained findings, both the UK and Singapore are potential targets for fishery export countries. However, the promotion strategies need to differ because food consumption is driven by different factors in each country.

The strength of this study is the use of consecutive sampling with proportional sample sizes. However, there are several limitations that need to be addressed. First, we could not rule out a recall bias related to the self-reported fish consumption. Second, the variable price is based on consumers’ perceptions rather than on actual values. Third, an internet survey can be subject to significant bias resulting from under-coverage and a lack of responses. However, we reduced this bias by using a random sample strategy, and we only included participants who completed the survey. Fourth, some of the questions asked about fish preferences in comparison with 10 years previously. Remembering events and habits of 10 years ago might be really challenging for some people; however, several studies [12,13,14] have used this technique to compare a current event with the one that occurred 10 years ago.

## Figures and Tables

**Table 1:** Summary of the survey questionnaire for multiple logistic regression.

	Variable Name	Question	Options
Dependent Variable	The desire of consuming fish	How has your desire to eat fish changed in comparison with 10 years ago?	1. It has increased 2. It has decreased 3. It has not changed
Independent Variables	The price of the fish	We would like to ask about your current situation in comparison with 10 years ago. Please select the closest answer for each item.	1. Closer to A (Fish has become cheaper) 2. Between A and B 3. Closer to B (Fish has become more expensive)
	Household income		1. Closer to A (Household income has increased) 2. Between A and B 3. Closer to B (Household income has decreased)
	Health related reasons (1)		1. Closer to A (I have started to think that eating fish is good for my health (e.g. omega fatty acids, nutrition)) 2. Between A and B 3. Closer to B (I have started to think that eating fish is bad for my health (e.g. organic mercury, dioxins) )
	Health related reasons (2)		1. Closer to A (I have started to think that eating meat is dangerous (e.g. mad cow disease, avian flu)) 2. Between A and B 3. Closer to B (I have started to think that eating meat is safe.)
	Fish variety		1. Closer to A (Fish variety has increased (e.g., more imported fish) ) 2. Between A and B 3. Closer to B (Fish variety has decreased (e.g., less imported fish))
	Sustainability concerns		1. Closer to A (The opportunity to purchase fish produced in sustainable fisheries has increased) 2. Between A and B 3. Closer to B (The opportunity to purchase fish produced in sustainable fisheries has decreased )
	Age	What is your age?	Age of respondent
	Sex	What is your gender?	1. Male, 2. Female
	Education	Your highest education level is:	1. Primary to Junior high school, 2. High school, 3. Vocational school and junior college, 4. University (Bachelors or 4-year degree), 5. Graduate school, 98. Other—Please specify:, 99. I decline to answer
	Having religion	Your religion is:	1. Hinduism, 2. Christianity, 3. Islam, 4. Buddhism, 5. Sikhism, 6. Judaism, 7. Taoism, 8. No religion, 98. Other—Please specify: , 99. I decline to answer
	Household composition	Please indicate your relationship to the people you are currently living with.	1. Spouse, 2. Child, 3. Grandson, 4. Parent or parent-in-law , 5. Brother/sister or brother/ sister-in-law, 98. Other—Please specify: , 99. Living alone

**Table 2:** The socio-demographic characteristics and dependent variables of the study population.

		Total	UK	Singapore	*p*-Value
		% (*n* = 1200)	% (*n* = 600)	% (*n* = 600)	
**Age (mean ± SD)**		**43.7 ± 13.4**	**44.1 ± 13.9**	**43.3 ± 12.9**	**<0.001**
Sex					0.82
	Male	49.3	49.7	49	
	Female	50.7	50.3	51	
Education					<0.0001
	Lower than high school	2.7	1.4	4	
	High school level	21.4	29.7	13.3	
	Vocational school level	23	23.9	22.2	
	Undergraduate level	42.1	37.1	47	
	Graduate level	10.8	7.9	13.5	
Religious					<0.001
	Yes	66.1	51.8	80.3	
	No	30.2	42.7	17.7	
	Unknown	3.8	5.5	2	
Household composition					<0.0001
	With family	87	79.5	94.5	
	Living alone	13	20.5	5.5	
The desire of consuming fish					<0.003
	Has increased	44	40.3	47.7	
	Has decreased	9.5	8.3	10.7	
	Unchanged	46.5	51.3	41.7	

**Table 3:** Multiple logistic regression of increased desire to consume fish.

		The UK		Singapore	
		Odds Ratio	(CI 95%)	Odds Ratio	(CI 95%)
Age					
	20–29 years old	6.8	(2.99–15.43)	1.43	(0.71–2.91)
	30–39 years old	2.15	(1.01–4.59)	1.76	(0.87–3.57)
	40–49 years old	1.92	(0.92–3.98)	1.26	(0.66–2.42)
	50–59 years old	1.7	(0.84–3.44)	1.34	(0.72–2.51)
	60–69 years old		1.00		1.00
Sex		0.72	(0.45–1.15)	0.95	(0.64–1.40)
The price of the fish					
	It has become cheaper	3.44	(1.71–6.93)	5	(2.12–11.77)
	Neutral		1.00		1.00
	It has become more expensive	1.2	(0.72–2.01)	1.3	(0.83–2.03)
Household income					
	It has increased	1.02	(0.54–1.93)	1.15	(0.66–1.99)
	Neutral		1.00		1.00
	It has decreased	1.48	(0.81–2.74)	0.84	(0.50–1.40)
Health related reasons (1)					
	Fish is good for health	3.67	(1.46–9.21)	0.42	(0.19–0.95)
	Neutral		1.00		1.00
	Fish is bad for health	0.95	(0.38–2.42)	0.35	(0.15–0.80)
Health related reasons (2)					
	Meat is unsafe to be consumed	2.82	(1.24–6.41)	1.24	(0.61–2.50)
	Neutral		1.00		1.00
	Meat is safe to be consumed	1.48	(0.81–2.74)	0.74	(0.39–1.40)
Fish variety					
	It has increased	1.25	(0.43–3.64)	1.82	(0.89–3.71)
	Neutral		1.00		1.00
	It has decreased	1.1	(0.38–3.19)	1.68	(0.85–3.31)
Sustainability concerns					
	Sustainable fisheries has increased	0.68	(0.27–1.73)	0.53	(0.25–1.10)
	Neutral		1.00		1.00
	Sustainable fisheries has decreased	0.56	(0.22–1.42)	1.49	(0.25–0.98)
Education					
	High school level	1.44	(0.87–2.36)	1.11	(0.71–1.74)
	Vocational school level		1.00		1.00
	Undergraduate level	1.84	(0.74–4.58)	1.9	(0.98–3.66)
Religious					
	Yes	1.03	1.01–1.05	0.95	(0.88–1.02)
	No		1.00		1.00
Household composition					
	With family	1.77	(0.97–3.22)	0.71	(0.31–1.63)
	Alone		1.00		1.00

## References

[1] The Fisheries Agency of Japan (2011). White Paper on Fisheries. http://www.jfa.maff.go.jp/j/kikaku/wpaper/h23/pdf/04_dai2shou.pdf.

[2] FAO Fisheries and Aquaculture Department (2010). The State of World Fisheries and Aquaculture. http://www.fao.org/docrep/013/i1820e/i1820e.pdf.

[3] Armitage C.J., Conner M. (2001). Efficacy of the theory of planned behaviour: A meta-analytic review. Br. J. Soc. Psychol..

[4] Verbeke W., Vackier I. (2005). Individual determinants of Fish Consumption: Application of the Theory of Planned Behavior. Appetite.

[5] Olsen S.O., Heide M., Dopico D.C., Toften K. (2008). Explaining Intention to Consume a New Fish Product: A Cross-Generational and Cross-Cultural Comparison. ‎Food Qual. Preference.

[6] Kaimakoudi E., Polymeros K., Schinaraki M.G., Batzios C. (2013). Consumers’ Attitudes Towards Fisheries Products. Proc. Technol..

[7] Brouwer A.M., Mosack K.E. (2015). Expanding the Theory of Planned Behavior to Predict Healthy Eating Behaviors: Exploring a Healthy Eater Identity. Nutr. Food Sci..

[8] Government Appointed Industry Expert Working Group Members (2017). Seafood 2040. http://www.seafish.org/media/publications/SEAFOOD_2040_lo_singlep.pdf.

[9] Roheim C.A., Asche F., Santos J.I. (2011). The Elusive Price Premium for Ecolabelled Products: Evidence from Seafood in the UK Market. J. Agric. Econ..

[10] Herrmann R.O., Rauniyar G.P., Hanson G.D., Wang G. (1994). Identifying Frequent Seafood Purchasers in the Northeastern US. Agric. Resour. Econ. Rev..

[11] (2017). Global Agriculture Information Network, Singapore: Seafood Report 2017.

[12] Jorm A.F. (1994). A short form of the Informant Questionnaire on Cognitive Decline in the Elderly (IQCODE): Development and cross-validation. Psychol. Med..

[13] Smith C.A., Henderson V.W., McCleary C.A., Murdock G.A., Buckwalter J.G. (2000). Anosognosia and Alzheimer’s Disease: The Role of Depressive Symptoms in Mediating Impaired Insight. J. Clin. Exp. Neuropsychol..

[14] Reilly W.T., Talley N.J., Pemberton J.H., Zinsmeister A.R. (2000). Validation of a Questionnaire to Assess Fecal Incontinence and Associated Risk Factors. Dis. Colon Rectum.

[15] Fox N., Ward K. (2008). Health, ethics and environment: A qualitative study of vegetarian motivation. Appetite.

[16] Nayga R.M., Capps O. (1995). Factors Affecting the Probability of Consuming Fish and Shellfish in the Away from Home and At Home Markets. J. Agric. Appl. Econ..

[17] Chopra A., Bessler D.A. Impact of BSE and FMD on beef industry in UK. Proceedings of the NCR-134 Conference on Applied Commodity Price Analysis, Forecasting, and Market Risk Management.

[18] Daniel M.C. (2002). Sociocultural Considerations of Fish Consumption. Comments Texicol..

[19] Assadi D. (2003). Do Religions Influence Customer Behaviour? Confronting Religious Rules and Marketing Concepts. Cahiers du CEREN.

